# All Hands on Deck: Nurses and Cancer Care Delivery in Women’s Health

**DOI:** 10.3389/fonc.2016.00174

**Published:** 2016-07-22

**Authors:** Jeanne Murphy, Michelle Mollica

**Affiliations:** ^1^Cancer Prevention Fellowship Program, Division of Cancer Prevention, National Cancer Institute, Rockville, MD, USA

**Keywords:** nursing, advanced practice nursing, oncology, roles

Access to expert gynecologic oncology care is hampered by geographic (1), racial (2), and socioeconomic disparities (3). As cancer care grows in complexity and expense (4) with an aging and increasingly diverse population, the Institute of Medicine and others have called for improvements in cancer care delivery and research (5, 6). The growing workforce gap in supply of gynecologic oncologists – where demand is increasing, but number of providers remains stagnant (2) – highlights the need for fully utilizing the skills of all clinicians working across the cancer control continuum (prevention, screening, treatment, survivorship, and end of life). To that end, nurses can have an enormous impact on improving and expanding access to oncology care as clinicians, designers, and leaders of initiatives to improve care. Nurses comprise the largest group of health-care providers in the U.S. (7). In its 2010 report on the future of nursing, the Institute of Medicine called for all nurses to practice to the full extent of their nursing “education, training, and competencies” (5). We argue that promoting and expanding nurses’ roles within innovative, multidisciplinary models of care in women’s health is essential in order to improve growing gaps in cancer care.

## Prevention

### Primary Prevention

Prevention of common women’s cancers includes promotion of healthy lifestyles and vaccination, though the potential for widespread dissemination is hampered by ineffective implementation. For example, the prophylactic human papillomavirus (HPV) vaccine is a groundbreaking prevention tool which is now available to prevent cervical cancer. Uptake of the vaccine among youth in the U.S. is inadequate despite widespread insurance coverage and availability. In 2014, <40% of U.S. teenagers completed the three dose series before 18 years of age (8). Although physician recommendation has been shown to improve uptake of HPV vaccines (9), the recommendations of nurses could be equally or more effective in increasing HPV vaccine uptake in primary care, especially in rural and underserved areas (10). Gallagher et al.’s systematic review demonstrated that, despite challenges, school-based HPV vaccination programs in the U.S. and other countries have achieved higher levels of vaccine uptake when compared with those conducted at health-care facilities (11). School nurses are integral to such programs, where qualitative research by Boyce and Holmes demonstrated that they have the potential to promote vaccination of medically underserved children (12). In particular, school nurses often serve as opinion leaders in middle schools – where the target population for HPV vaccination is found – and can intervene with targeted education, follow-up, and tool kits to promote vaccination among students and parents (13, 14).

### Health Education

Advanced practice nurses, such as nurse practitioners (NPs) and certified nurse midwives (CNMs), are often the only source of primary health care for women, especially in medically underserved areas (15). A major focus of NP and CNM practice is health education and promotion of healthy behaviors (16, 17). NPs have led successful cardiovascular disease interventions in smoking cessation (18, 19) and obesity prevention (20) which would have crossover benefits for cancer prevention in the long term, and their contributions should be utilized to maximize prevention efforts for women.

## Screening

### Early Detection

Nurses have many opportunities to reduce the substantial gaps in access to gynecologic and breast cancer screening in the U.S., particularly for minority and underserved women (21). NPs and CNMs play important roles in providing primary care by performing cervical cancer screening, referring women for mammography and colon cancer screening, and then collaborating with or transferring care to specialist physicians as necessary (22). Despite their education, training, and evidence that their quality and patient satisfaction outcomes are equal or superior to that of physicians (7, 16, 23), NPs and CNMs are still underutilized in extending the reach of cervical and breast cancer screening in underserved communities (24, 25).

### Navigation

Nurses may also work in a navigation role in primary care practice, helping patients understand the importance of cancer screening and follow-up after abnormal results. As an example, nurse navigation demonstrated an increase in women’s follow-up colposcopy attendance after abnormal cytology screening (26). Utilizing all available nursing professionals in ambulatory settings would provide the comprehensive approach needed to improve cancer prevention care for women on a broad population level.

## Diagnosis and Treatment

As integral members of the cancer care team during treatment, nurses’ involvement in multidisciplinary cancer care treatment models can improve care post-diagnosis through management of treatment-related symptom toxicities, and improving adherence to treatment.

### Symptom and Toxicities Management

Treatment-related symptom toxicities, particularly in novel therapies such as targeted agents and immune therapies, are often serious but difficult to recognize, and thus likely under reported (27). Nurses can play an integral role in the integration of patient-reported outcomes related to such therapies, particularly during assessment, patient education, and through communication *via* patient portals. The Oncology Nursing Society outlines competencies for certification of oncology nurses and NPs (28). Oncology Certified Nurses (OCN) are trained to provide specialized patient care that is validated by certification of knowledge in oncology nursing focusing on adults with cancer (29). Oncology NPs are specialists in symptom management (30) and discharge planning to improve quality of life long term.

In addition, nurses and NPs can offer support and education around discussions about sexual health (31) and fertility preservation (32). Nurses at the bedside or on research teams are essential for effective recruitment of women with gynecologic cancer to the clinical trials needed to improve cancer-related care (33) and have held leadership positions on gynecologic cancer treatment trials (34). Utilization of specially qualified nurses in many roles can enhance both the reporting and subsequent treatment of treatment-related symptoms and complications.

### Adherence

Cancer centers increasingly utilize nurse navigators to assist women through complicated care regimens (35–37), resulting in increased adherence to treatment (38) and improved patient satisfaction with care (39). In addition, geographic differences can play a role in cancer treatment outcome disparities (1, 40). Oncology NPs can increase access to care for underserved areas through telemedicine and satellite clinics, addressing issues with access and facilitating appointment adherence (28). Innovative multiprovider visits, including medical and nursing staff, have been designed for women who are initiating ovarian cancer chemotherapy. Prescott et al. (41) described a shared medical visit model in which a multidisciplinary team, including the oncologist, NPs, nurses, and social workers, provided standardized education visits for gynecologic oncology patients planning to begin their series of platinum-based chemotherapy sessions. Nurses were integral in educating patients on expected side effects, coping tools, and the importance of shared decision-making throughout treatment. Nurse-led support groups can be important outlets for patients to support adherence through difficult treatment regimens.

Adjuvant hormone therapy, including tamoxifen and aromatase inhibitors, is a widely recognized and important component of breast cancer treatment for hormone receptor positive women. Despite the documented benefits, up to 50% of women who are recommended therapy do not initiate therapy or do not adhere to the regimen for the recommended 5–10 years, due in part to the myriad of side effects of hormonal treatment (42). In addition, as many cancer therapies move from intravenous to oral medications with complex home regimens, adherence becomes an increasingly important area where nurses can improve outcomes. Schneider et al. (43) described a small clinical trial (*N* = 45) of tailored nursing education intervention which improved both self- and pharmacy-reported adherence to oral chemotherapy (93% in intervention vs. 80% in controls at 2 months, no CI given). Nurses should play a key role in increasing patient knowledge of side effects and remedies, communicating benefits of treatment to prevent recurrence, and identifying coping strategies to resolve barriers to adherence.

## Survivorship

There is a growing need to address the many late and long-term effects that plague the growing number of gynecologic cancer survivors (44), and nurses at all levels are integral in this care.

### Navigation Posttreatment

While appropriate utilization and implementation of survivorship care plans are still being explored (45, 46), nurse navigators coordinate care as the cancer patient transitions back to primary care after active treatment. In addition, primary care, oncology, and advanced practice nurses educate the patient throughout treatment and into survivorship on managing the transition after a cancer diagnosis, including late and long-term effects, as well as the on importance of follow-up care after treatment to detect recurrence or secondary malignancies (47).

### Clinical Care of Survivors

Models of care delivery for survivorship care include primary care, gynecologic oncologist-led, and survivorship clinics, offering multidisciplinary services. While there are differing opinions on the best setting for long-term follow-up and care of survivors, and this may differ based on cancer type and individual provider or institutions, there is emerging research that nurse-led survivorship clinics hold potential for this important care (48, 49). A technical report on models of survivorship care indicated that cancer survivors preferred follow-up from those with specialized training (50), and pointed to the need for more specialized survivorship training for oncology nurses and advanced practitioners. In addition, a systematic review comparing models of survivorship care for posttreatment follow-up of adult cancer survivors found no significant differences in quality of life or disease recurrence outcomes for nurse-led follow-up when compared with oncologist-led follow-up care. In fact, patient satisfaction was higher for nurse-led care in one study included in the review (51). Rosenberg and colleagues (52) explored the use of survivorship risk-adapted follow-up visits facilitated by an oncology nurse and involving discussion of survivorship care plans. The authors found that of the 1615 breast cancer survivors who participated in the intervention, most reported more confidence in understanding their diagnosis, treatment summary, and recommendations for posttreatment support. Overall, as nurse-led clinics are typically less costly to an organization, specialized nurses working in consultation with physicians could increase availability of oncology survivorship services.

## End-of-Life Care

When a patient’s prognosis changes and goals of curative treatment transition into advanced care planning, nurses can uniquely contribute in many areas. Advanced practice nurses in both oncology and primary care settings should be trained to effectively communicate conversations about worsening prognosis (53); however, application of this role is unclear in the literature; this represents a missed opportunity for improving an essential aspect of cancer care. Oncology nurses in both inpatient and outpatient settings establish strong relationships with patients through many hours of patient contact and can play a substantial role in helping patients and families consider their own goals and values as they relate to end-of-life care. Research indicates, however, that nurses experience ethical dilemmas surrounding these conversations, as their role is less defined and they are often hesitant to have these conversations with patients and families (54, 55). Inpatient nurses, who could be trained in end-of-life care, are able to function as “champions,” for example, and act as a resource for their team (56). While physician–nurse teams are optimal for discussions surrounding end-of-life care, more education and training for nurses are important in order to optimize communication with advanced cancer patients as their prognosis worsens.

## Conclusion

Women diagnosed with gynecologic cancers face difficult and complex treatment regimens with long-term health implications. Innovations and improvements in cancer care delivery in women’s health must rely on all members of the health-care team. We argue that because the nursing workforce is vastly larger than that of gynecologic oncologists, and boasts breadth and depth of roles, training, and capabilities, it is essential to better utilize and integrate nurses within the multidisciplinary team to ensure comprehensive, woman-centered care before, during, and after cancer. It is time that women had equitable access to higher quality cancer care, and nursing is up to the challenge.

## Author Contributions

JM and MM wrote early drafts and edited the final draft of the article.

## Conflict of Interest Statement

The authors declare that the research was conducted in the absence of any commercial or financial relationships that could be construed as a potential conflict of interest. The handling editor declared a shared affiliation, though no other collaboration, with the authors and states that the process nevertheless met the standards of a fair and objective review.
